# Urinary calcium excretion in children with monosymptomatic enuresis

**DOI:** 10.1007/s11845-014-1217-x

**Published:** 2014-10-30

**Authors:** A. Korzeniecka-Kozerska, T. Porowski, A. Wasilewska, M. Stefanowicz

**Affiliations:** Department of Pediatrics and Nephrology, Medical University of Bialystok, 17 Waszyngtona Street, 15-274 Bialystok, Poland

**Keywords:** Calcium excretion, Hypercalciuria, Ionized calcium, Monosymptomatic enuresis, Urinary calcium

## Abstract

**Background:**

Among many factors predisposing to monosymptomatic enuresis (MNE) disturbances in urinary electrolites excretion play an important role. Because of many controversies in this field there is a need to debate the role of hypercalciuria in MNE. The aim of our study was to determine the urinary calcium in children with MNE.

**Methods:**

The investigation was conducted on 204 children (83 MNE children and 121 reference group). Urinary calcium excretion (in 24-h collection and per kg of body mass), Ca/creatinine ratio, Ca^2+^ in urine sample and in 24-h collection of urine were estimated.

**Results:**

Hypercalciuria in MNE group was diagnosed in 18/83 (21.69 %) patients. We found statistically significant differences between children with MNE in Ca^2+^ in urine sample and 24-h collection and Ca/creat. ratio. Median urinary calcium excretion (mg/kg/24-h and mmol/24-h) was significantly higher in hypercalciuric enuretic patients. The urinary total calcium (mmol/24-h), urinary bound calcium and urinary calcium concentration (mmol/L) demonstrated a significant positive correlation with height, weight and age in reference group but not in MNE group.

**Conclusion:**

Urinary calcium excretion was significantly disturbed and further studies are needed to assess the role of hypercalciuria in the pathogenesis of MNE.

## Introduction

Among many factors predisposing to nocturnal enuresis disturbances in urinary electrolites excretion play an important role. It is well-known that patients presenting nocturnal polyuria have increased nocturnal natriuria and low vasopressin level [1, 2]. Pharmacological treatment recommended in monosymptomatic enuresis (MNE) based on antidiuretic hormone increases excretion of Ca^2+^ [3]. There are some studies on disturbed calcium-phosphate balance in children with MNE. It is worth mentioning that nocturnal hypercalciuria (HC) was observed in patients either with nocturnal polyuria or without [4–6]. Pace et al. [7] and Valenti et al. [8] diagnosed hypercalciuria both in patient with normal fluid balance and in patients with nocturnal polyuria and stated that enuresis can be caused by absorptive hypercalciuria. In contrast to those studies, Kamperis et al. [9] claim that there are no differences in calcium excretion between enuretic individuals with and without nocturnal polyuria [10].

To our best knowledge hypercalciuria can be associated with such signs and symptoms as: dysuria, nocturnal and/or diurnal incontinence and urge incontinence which are improved after success treatment of hypercalciuria and relapsed after interruption or reducing medication [11, 12].

Because of many controversies in this field there is a need to continue debate about the role of hypercalciuria and new strategies for the management of MNE.

The aim of our study was to determine the urinary calcium in children with monosymptomatic enuresis.

## Methods

The investigation was conducted on 204 children. The study group included 83 MNE children with the median age of 9.66 years. Studied group was further divided into two groups according to urinary calcium excretion: the first (normocalciuric children with monosymptomatic enuresis-MNEN) was comprised of 65 patients with the median age of 10.01 years and the second one (hypercalciuric children with monosymptomatic enuresis-MNEH) was composed of 18 children with median age 7.43 years. The diagnostics of HC was based on the urinary excretion of calcium equal or higher than 4 mg/kg/24 h [13, 14]. MNE is defined as enuresis in children without any other lower urinary tract symptoms (LUTS) (nocturia excluded), and without previous history of bladder dysfunction [15]. Both groups of children were followed up in the Department of Pediatrics and Nephrology (2007–2012). Only non pharmacological treatment was applied with no improvement.

The reference group consisted of 121 children with the median age of 9.99 years. The urine samples from the reference group were obtained from children, mainly volunteers with a past history of inguinal hernia who had been approached to participate while attending to academic center or the children of hospital staff. None of the controls reported a family history of disturbed calcium balance.

### Inclusion criteria

(1) Patients aged 4–17 years with MNE, (2) completed 24-h urine collection, (3) normal renal function (eGFR > 90 ml/min/1.73 m^2^), serum creatinine and calcium level.

### Exclusion criteria

(1) Urinary tract infections in anamnesis, secondary nocturnal enuresis, LUTS, (2) signs of acute infection within 2 months before examination, (3) any pharmacological treatment or diet supplementation in past 6 months (calcium, Vitamin D), (4) any endocrinopathies, nephropathies, urinary tract or metabolic diseases, (5) inadequate 24-h urine collection.

The clinical work up included: collecting data to determine age, gender, previous treatment, physical examination, measurement of height, weight and BMI in all participants.

The biochemical work up included: serum creatinine (measured by Jaffe Gen. 2, Cobas Integra 800, Roche), urea, GFR (ml/min/1.73 m^2^) estimated by the Counahan-Barratt Equation (eGFR): GFR = 0.43 × L (cm)/Scr (mg/dl), L-length, Scr-serum creatinine level.

The urinary calcium (uCa) work-up included: urinary calcium excretion (in 24-h collection and per kg of body mass), Ca/creat. ratio (normal value less than 0.21), Ca^2+^ in urine sample and in 24-h collection.

The urine was aseptically collected between 7 and 8 am from the morning sample. To prevent urine loss the enuretic children were waking up two times during night. The completeness of the 24-h urine collections was evaluated by comparing the total creatinine in the sample with the predicted creatinine (normal range 15–25 mg/kg/24 h). Urinary tract infections were excluded on the basis of urinary testing. Current infection was excluded based on the negative C-reactive protein (CRP).

### Methods

The 24-h urine samples were stored at a temperature of +4 °C, without preservatives. All measurements were conducted within 4-h after collection.

Urinary calcium and creatinine were assessed with the Cobas-Integra 800 (Roche, Indianapolis, IN). The urinary Ca^2+^ concentration was measured using calcium ion-selective electrodes (Rapidlab 855; Bayer, Leverkusen, Germany). Calibration and the quality assurance procedure, based on the calibration curves, were carried out every day. The corresponding value for urinary bound Ca was calculated as the difference between Ca total and Ca^2+^. Urinary pH was determined using a microcomputer pH meter (model CP-315 M; Elmetron, Zabrze, Poland).

Data analysis was performed using Statistica ver. 10.0 (StatSoft Inc., Tulsa, OK, USA). Normal distribution of data was tested with the Shapiro–Wilk *W* test and then statistical analysis was performed using non-parametric tests (Mann–Whitney and Spearman). A *p* value <0.05 was considered statistically significant.

Written informed consent was obtained from all the enrolled subjects, subsequent to receive full information about the study.

The study was approved by the Ethics Committee of the Medical University of Bialystok in accordance with the Declaration of Helsinki.

## Results

Hypercalciuria in MNE group was diagnosed in 18/83 (21.69 %) patients. The basic characteristics of the study subjects are presented in Table 1. There were no differences in the age (*p* = 0.899), height (*p* = 0.272), weight (*p* = 0.246), BMI (*p* = 0.269) between enuretic children and the reference group. Groups were also sex-matched. Serum creatinine concentration and urinary creatinine excretion (g/24 h) did not differ significantly among MNE patients and the reference group (*p* = 0.692). MNEN and MNEH children differed from controls with respect to UCa bounded (*p* = 0.02 and *p* < 0.01, respectively) and to uCa concentration (mmol/L) (*p* = 0.01 and *p* < 0.01, respectively). Statistically significant differences in these parameters were found between MNEN and MNEH patients (*p* < 0.01).

**Table 1:** The median values and ranges of basic demographical data and examined parameters in ME, MEN, MEH and reference group

Parameters (SI)	Median (min–max)			
	ME	MEN	MEH	Reference group
Age (years)	9.66 (4.16–16.98)	10.01 (4.16–16.98)	7.43 (4.73–15.01)	9.99 (4.15–16.86)
Height (cm)	137.5 (107–188)	139 (107–188)	125 (113–173.5)	143 (108–191)
Weight (kg)	32 (12.1–111)	32 (17–111)	23.75 (12.1–63)*	36 (16–113.4)
BMI (kg/m^2^)	16.16 (9.48–31.41)	16.22 (13.04–31.41)	15.87 (9.48–21.05)	17.52 (12.21–31.58)
S-crea. (mg/dl)	0.51 (0.28–0.91)	0.53 (0.28–0.91)	0.44 (0.31–0.75)	0.49 (0.31–0.93)
U-crea. (g/24 h)	0.58 (0.29–2.08)	0.61 (0.31–2.08)	0.47 (0.29–1.52)	0.73 (0.32–1.88)
U Ca^2+^ (mmol/L)	0.44 (0.01–1.97)**	0.33 (0.01–0.81)	1.14 (0.76–1.97)**	0.28 (0.12–0.82)
U Ca^2+^ (mmol/24)	0.33 (0.01–1.87)**	0.26 (0.009–1.3)	0.74 (0.3–1.87)**	0.22 (0.05–0.9)
Ca/creat. ratio (mg/mg/24 h)	0.11 (0.01–0.78)*	0.09 (0.01–0.2)	0.3 (0.13–0.78)**	0.09 (0.02–0.196)
eGFR (ml/min/1.73 m^2^)	133.88 (91–316.64)*	125.52 (91–316.64)	150.26 (100.99–260)	143.3 (92.19–222.33)
24-h urine collection (ml)	750 (350–2,700)	800 (350–2,700)	647 (400–1,350)	700 (250–2,300)
24-h urine collection (ml/kg)	25.53 (6.58–72.06)*	25 (6.58–72.06)	25.53 (13.33–40.48)	20.53 (5.91–62.16)
Ca (mg/kg/24-h)	2.06 (0.l6–10.58)	1.76 (0.16–3.87)	4.83 (4.28–10.58)**	1.7 (0.37–3.48)
Ca (mmol/24-h)	1.58 (0.24–9.87)	1.398 (0.24–6.5)	3.18 (1.37–9.87)**	1.51 (0.27–4.48)
U Ca bounded (mmol/24-h)	1.27 (0.1–8.0)	1.08 (0.1–5.24)*	2.37 (0.91–8.0)**	1.27 (0.15–4.15)
U Ca total (mmol/L)	2.23 (0.23–10.39)	1.56 (0.23–4.35)*	4.85 (3.29–10.39)**	2.16 (0.34–8.58)
pH of urine	6.25 (5.44–7.68)	6.21 (5.44–7.68)	6.29 (5.81–7.0)	6.35 (5.43–6.32)
Osmolality (mOsm/kgH_2_O)	637 (226–1,212)*	625 (226–1,212)	711 (511–1,088)**	565 (240–1,135)

Comparison between studied groups and controls

*ME* patients with monosymptomatic enuresis, *MEN* normocalciuric patients with ME, *MEH* hypercalciuric patients with ME, *S-crea.* serum creatinine, *U-crea.* urinary creatinine, *U Ca* ^*2+*^ urinary calcium^2+^, *U Ca* urinary calcium, *eGFR* Counahan-Barratt Equation

* *p* < 0.05

** *p* < 0.01

No statistically significant differences were found in urine pH between patients and controls, similarly no difference was found between amount of 24 h urine collection (ml) in MNE children and the controls (*p* = 0.254). The median 24 h urine production (ml/kg body mass) was higher in MNE children than in reference (*p* = 0.034). There were no differences in this parameters between MNEN and MNEH patients (*p* = 0.669).

We found statistically significant differences between children with MNE in such parameters as: Ca^2+^ in urine sample and 24 h collection (*p* = 0.001), Ca/creatinine ratio (*p* = 0.04). We found increased levels of these parameters in MNEH patients but not in MNEN compared to controls (Ca^2+^ in urine sample: *p* < 0.01; *p* = 0.471, respectively; Ca^2+^ in 24 h urine collection: *p* < 0.01; *p* = 0.141, respectively, Ca/creat. ratio: *p* < 0.01; *p* = 0.588, respectively).

Median uCa excretion (mg/kg/24 h) was significantly higher in MNEH patients when compared to healthy controls (*p* < 0.01) and there were no differences between MNE children (*p* = 0.152) and MNEN children (*p* = 0.157) when compared to reference group. Median uCa excretion (mmol/24 h) was higher in MNEH children compared to healthy individuals (*p* < 0.01). We did not observe differences in this parameter between MNE and MNEN compared to reference group (*p* = 0.575 and *p* = 0.096, respectively).

Additionally, we analyzed urine osmolality (24 h collection). Median osmolality MNE children (637 mOsm/kgH_2_O) was higher than in reference group (565 mOsm/kgH_2_O) (*p* = 0.02). We found statistically significant differences between urine osmolality in MNEH (711 mOsm/kgH_2_O) vs. reference group (*p* = 0.004) but not between MNEN (625 mOsm/kgH_2_O) vs. reference group (*p* = 0.139).

The urinary total calcium (mmol/24 h), urinary bound calcium and urinary calcium concentration (mmol/L) demonstrated a positive significant (*p* < 0.05) correlations with height (*R* = 0.5332; *R* = 0.5774; *R* = 0.276, respectively), weight (*R* = 0.5111; *R* = 0.5545; *R* = 0.2280, respectively) and age (*R* = 0.5502; *R* = 0.6001; *R* = 0.3171, respectively) in reference group but not in MNE group. We found statistically significant positive correlation (*p* < 0.05) between Ca^2+^ (mmol/24 h) vs. height (*R* = 0.5725), weight (*R* = 0.6694) and age (*R* = 0.4799) in MNEH group but not in reference. Statistically significant negative correlations (*p* < 0.05) was revealed between urine Ca^2+^ (mmol/L) and height (*R* = −0.2296), weight (*R* = −0.3059) and age (*R* = −0.2197) in MNE group but not in reference. The Ca/creat. ratio and uCa amount (mg/kg/24 h) demonstrated negative correlations with height (*R* = −0.2349; *R* = −0.2685, respectively), weight (*R* = −0.3178; *R* = −0.3791) and age (*R* = −0.2491; *R* = −0.2767, respectively) in MNE group.

**Fig. 1:**
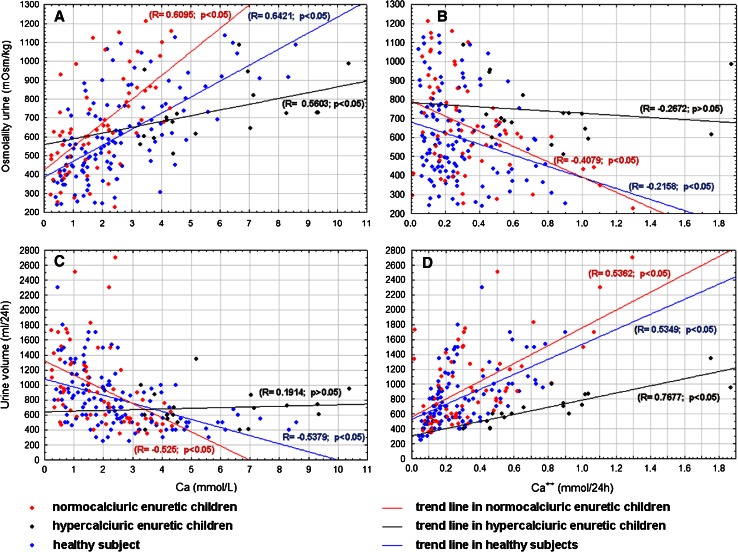
Correlations between calcium excretion [Ca concentration (mmol/L) and Ca^2+^ (mmol/24 h) and osmolality (**a**, **b**) and urine volume (**c**, **d**) in all studied group]

**Fig. 2:**
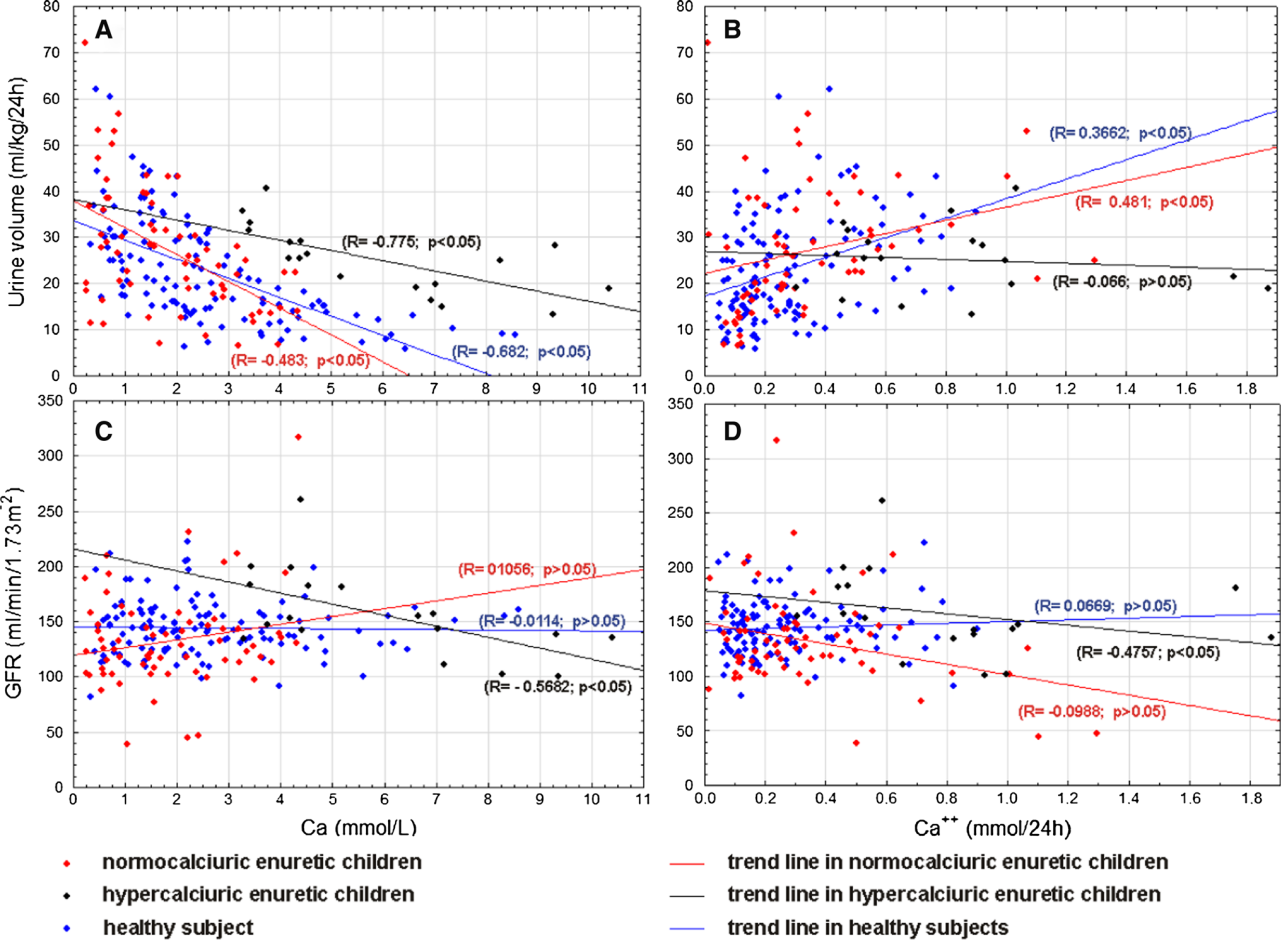
Correlations between calcium excretion [Ca concentration (mmol/L) and Ca^2+^ (mmol/24 h) and urine volume (**a,** **b**) and GFR (**c**, **d**) in all studied group]

Correlation between uCa excretion and osmolality, 24 h urine collection and eGFR in all studied group are presented in Figs. 1 and 2 and additionally in Table 2.

**Table 2:** Correlations between parameters of calcium excretion and urine osmolality and daily urine collection in children with monosymptomatic enuresis (ME)

	Osmolality (mOsmol/kgH_2_O)		24-h urine collection (ml/kg body mass)		24-h urine collection (ml)	
	MEN	MEH	MEN	MEH	MEN	MEH
	*R*	*R*	*R*	*R*	*R*	*R*
U Ca (mmol/24 h)U	−0.152	0.040	−0.017	−0.512*	0.1497	0.815*
U Ca bounded (mmol/24 h)	−0.08	0.178	−0.171	−0.617*	0.0225	0.700*
U Ca/crea (mg/mg/24 h)	−0.036	−0.0872	0.2597*	0.1559	0.12	0.39
U Ca/kgb.m./24 h	0.125	0.112	0.197	0.0299	−0.02	0.267
U Ca^2+^ (mmol/L)	−0.253*	0.0722	0.059	−0.1585	−0.13	0.502*

*MEN* normocalciuric children with ME, *MEH* hypercalciuric children with ME, *U Ca* urinary calcium

Significant value: * *p* < 0.05; ** *p* < 0.01

## Discussion

In the first study of its kind, our study comprehensively describes uCa excretion, particularly based on total calcium, bound calcium and free calcium (Ca^2+^) in children with MNE.

The results of our investigations show that more than 20 % of MNE children from our centre presented with hypercalciuria and exhibited significantly higher calcium excretion in comparison with the reference group. Similar results were confirmed by Vlavi et al. [16] who found hypercalciuria in 21.3 % of enuretic children. The prevalence of hypercalciuria in healthy children has been reported to be between 3 and 7 % [10]. To our best knowledge, hypercalciuria is observed in all age groups, regardless of gender and race [17, 18]. The results of our study indicated that uCa excretion does not differ between girls and boys. Penido et al. [11] showed that hypercalciuria was present in all age groups but most prevalent in school-age children. According to the literature, age-dependent decrease in prevalence of enuresis can be caused by maturation of central nervous system [19]. Is it possible that an increased rate of spontaneous remission could be caused by normalization of calciuria depending on age? It is well-known that successful treatment of hypercalciuria resolves all abnormal LUTS [20, 21]. An interesting, novel finding based on our research was that there was a negative correlation observed between calcium excretion and age in the MNE group. In the reference group, calciuria correlated positively with age, height and weight, but calcium excretion did not exceed the normal range. To confirm uCa excretion, we estimated uCa (mg/kg body mass), bound calcium and Ca^2+^ concentration, Ca/creat. ratio, the amount of calcium during a 24-h urine collection, calcium concentration in the 24-h urine collection and compared all these parameters to the reference group.

The urinary Ca^2+^ measurement is not a common practice in children with MNE. However, some studies have estimated Ca^2+^ excretion in MNE children treated with desmopressin. In one such study, Muller et al. [3] described increased urinary Ca^2+^ excretion in enuretic children treated with desmopressin. The findings are never explicit and Vlavi et al. [16] concluded that hypercalciuria does not have a significant association with desmopressin therapy in enuretic children. These observations have led us to consider the need for estimation Ca^2+^ excretion before desmopressin treatment to exclude the desmopressin influence on Ca^2+^ excretion. We observed statistically significant differences in Ca^2+^ excretion in our MNE patients before any treatment compared to the reference group. In our investigations, significant differences were observed between MNE children and the reference group (*p* < 0.01) regarding Ca^2+^ excretion (both concentration and in 24 h collection).

Activation of detrusor muscle requires influx of extracellular Ca^2+^ through calcium channels as well as a mobilization of intracellular Ca^2+^ [22]. If increased Ca^2+^ concentration is observed, detrusor overactivity would be expected. This conclusion needs to be confirmed by an objective urodynamic study which we plan to conduct in the future. It seems that the estimation of Ca^2+^ excretion could be an attractive way of choosing proper diagnostics and therapy. However, further investigations are needed to clarify if MNEH patients present detrusor overactivity more often than healthy individuals or MNEN patients.

Sarici et al. [4] reported that enuretic children have significantly reduced bone mineral density and present retarded skeletal maturation but the exact mechanism responsible for this remains to be determined. Our findings add some clarity as to why enuretic children have problems with mineral density of bones. Hypercalciuria observed in patients with MNE can be responsible for disturbed bone mineralization and, additionally, can cause disturbed lower urinary function. Numerous authors have described these phenomena [23–26].

It was documented by Raes et al. [27] that urinary Ca/creat. ratio had shown significant linear regression with nocturnal diuresis rate and correlated with osmolar excretion normalized for urinary creatinine. We were not able to confirm this observation in our study. The Ca/creat. ratio showed negative correlation with osmolality and positive with urine volume but the findings were not statistically significant. A statistically significant positive correlation was found between osmolality and calcium concentration in urine in the MNEH and MNEN group and negative between Ca^2+^ (mmol/L) and Ca^2+^ (mmol/24 h) only in the MNEN group. The correlations observed in enuretic children may lead to inaccurate diagnoses of MNE children because patients with good osmolality caused by hypercalciuria instead of by normal ability of urine concentration could be excluded.

Another aspect of our study was to examine the correspondence between Ca^2+^ and/or calciuria in pediatric diseases. Recent data indicate increased Ca^2+^ concentration in stone-formers [28]. The urinary concentration of Ca^2+^ is largely pH-dependent. Urinary Ca^2+^ and pH are inversely related [29, 30]. Although we did not observe differences in the pH of urine between all studied groups, the differences in calcium excretion observed between the enuretic and reference group were so clear and statistically significant that they can suggest that patients with MNE may be in danger of forming kidney stones due to disturbed calcium balance and recommended fluid restrictions. Further investigations are needed to explain the correlations between hypercalciuria and the threat of stone formation.

Our study has some limitations. First, we did not perform urodynamics on our patients to assess bladder function, both during day and nighttime, and to correlate results with uCa excretion. To present, these investigations are not recommended in MNE. However, it seems that in spite of the invasiveness of urodynamics the benefits from performing this examination can exceed the risks and could be very useful in diagnosing children with MNE. Another limitation is inability to estimate effects of different treatments on hypercalciuria. Thus, further studies are necessary to clarify relationships between bladder function and hypercalciuria in MNE children before and after initial treatment. This is important so that recommendations for increased water intake and adjustments to the diet can be made and followed.

In summary, urinary calcium estimation can be used to exclude patients with hypercalciuria, which can be responsible for enuresis, through the influence it has on bladder function. It seems that MNE patients with hypercalciuria first should be identified and initial treatment for hypercalciuria should be applied before any interventions. A referral for urodynamic investigation could be considered in all hypercalciuric patients with monosymptomatic enuresis. A high percentage of hypercalciuria in ME individuals indicates that changes in the diagnostic plan may be required. So, it is worth measuring uCa calcium excretion in all MNE children for the purposes of early detection and choosing useful therapy to help enuretic children resolve these unpleasant symptoms.

## Conclusions

Urinary calcium excretion was significantly disturbed in patients with enuresis and further studies are needed to assess the role of hypercalciuria in the pathogenesis of MNE in children.
